# Parallel worlds and mixed economies: multi-proxy analysis reveals complex subsistence systems at the dawn of early farming in the northeast Baltic

**DOI:** 10.1098/rsos.230880

**Published:** 2023-10-04

**Authors:** Ester Oras, Mari Tõrv, Kristiina Johanson, Eve Rannamäe, Anneli Poska, Lembi Lõugas, Alexandre Lucquin, Jasmine Lundy, Samantha Brown, Shidong Chen, Liivi Varul, Vanda Haferberga, Dardega Legzdiņa, Gunita Zariņa, Lucy Cramp, Volker Heyd, Michaela Reay, Łukasz Pospieszny, Harry K. Robson, Kerkko Nordqvist, Carl Heron, Oliver E. Craig, Aivar Kriiska

**Affiliations:** ^1^ Institute of Chemistry, University of Tartu, Ravila 14 a, 50411 Tartu, Estonia; ^2^ Institute of History and Archaeology, University of Tartu, Jakobi 2, 51005 Tartu, Estonia; ^3^ Swedish Collegium for Advanced Study (SCAS), Linneanum, Thunbergsvägen 2, 75238 Uppsala, Sweden; ^4^ Department of Geology, Tallinn University of Technology, Ehitajate Tee 5, 19086 Tallinn, Estonia; ^5^ Archaeological Research Collection, Tallinn University, Rüütli 10, 10130 Tallinn, Estonia; ^6^ BioArCh, Department of Archaeology, University of York, Environment Building, Wentworth Way, YO10 5DD York, UK; ^7^ Institute for Archaeological Sciences, Department of Geosciences, University of Tübingen, Rümelinstrasse 23, 72070 Tübingen, Germany; ^8^ School of Humanities, Division of History, Tallinn University, Narva rd 25, 10120 Tallinn, Estonia; ^9^ Institute of Latvian History, University of Latvia, Kalpaka blvd 4, LV-1050 Riga, Latvia; ^10^ Department of Anthropology and Archaeology, University of Bristol, 43 Woodland Road, BS8 1UU Bristol, UK; ^11^ Department of Cultures, University of Helsinki, Unioninkatu 38, 00014 Helsinki, Finland; ^12^ Organic Geochemistry Unit, School of Chemistry, University of Bristol, BS8 1TS Bristol, UK; ^13^ Institute of Archaeology, University of Gdańsk, ul. Bielańska 5, 80-851 Gdańsk, Poland; ^14^ Helsinki Collegium for Advanced Studies, University of Helsinki, Fabianinkatu 24, 00014 Helsinki, Finland; ^15^ Department of Scientific Research, The British Museum, WC1B 3DG London, UK

**Keywords:** early agriculture, neolithic transition, ancient diet, biomolecular archaeology, organic residue analysis, stable isotope analysis

## Abstract

The transition from foraging to farming was a key turning point in ancient socio-economies. Yet, the complexities and regional variations of this transformation are still poorly understood. This multi-proxy study provides a new understanding of the introduction and spread of early farming, challenging the notions of hierarchical economies. The most extensive biological and biomolecular dietary overview, combining zooarchaeological, archaeobotanical, dietary stable isotope and pottery lipid residue analyses is presented, to unravel the nature and extent of early farming in the 3rd millennium cal BCE in the northeast Baltic. Farming was introduced by incoming Corded Ware cultural groups (CWC), but some dietary segregation existed within these communities, with some having more access to domesticates, others incorporating more wild resources into their diet. The CWC groups coexisted in parallel with local hunter–fisher–gatherers (HFG) without any indication of the adoption of domesticates. There was no transition from foraging to farming in the 3rd millennium cal BCE in the NE Baltic. Instead, we see a complex system of parallel worlds with local HFGs continuing forager lifeways, and incoming farmers practising mixed economies, with the continuation of these subsistence strategies for at least a millennium after the first encounter with domesticated animals.

## 1. Introduction

The introduction of farming has been considered one of the key elements of ancient subsistence changes and human–animal interactions. Recent research has disputed the notion of universal and linear economic change [1–6], highlighting the importance of deeply contextualized regional studies for revealing the diversity by which people ‘transitioned’ or adopted their subsistence strategies.

The roots of European agriculture go back to the Levant some 11 000 years ago, where it emerged from the local adoption of some native plants and animals, a process lasting more than a millennium [7,8]. The spread of early farming to other parts of Europe has been explained through the exchange of ideas (cultural diffusion) or population replacement [9]. The arrival of farming in the east Baltic (nowadays Lithuania, Latvia, Estonia, Finland) has been directly related to the latter, i.e. arrival of Corded Ware cultural groups (CWC) in the 3rd millennium cal BCE [10–16], with their migratory steppe-related ancestry [17–19] and burial customs, as well as material culture differing from the local hunter–fisher–gatherer (HFG) populations [20–23]. Yet, the biological legacy and the aftermath of the arrival of early farming in the east Baltic, set in the context of local forager communities, northern climatic conditions with dense woodland and aquatic landscapes, abundant wild resources and missing indigenous species suitable for domestication, has remained ambiguous. Recent biomolecular findings have confirmed that the introduction of new, domesticated species took place from the 3rd millennium cal BCE onwards [6,24–26], but we lack clarity concerning the extent and character that the subsistence and gross diet actually changed and for whom.

The goal of this study is to disentangle the complexity of early farming livelihood among CWC groups and assess its impact on local HFG populations in northern latitudes beyond the 56th parallel north, focusing on Estonian and Latvian (labelled as northeast Baltic from here onwards) 3rd millennium cal BCE material. To do so, we bring together an exceptional combination of a new bioarchaeological dataset covering human bone collagen stable isotope and pottery lipid residue analyses, zooarchaeological records together with recent direct accelerator mass spectrometry (AMS) radiocarbon dates, as well as archaeobotanical information from previous studies. Our findings show multifaceted regional adaptive strategies revealing both segregated dietary practices and mixed economy during the introductory phase of early farming, clearly deviating from the grand-scale narratives of linear and hierarchical models of ancient subsistence change.

**Figure 1.**
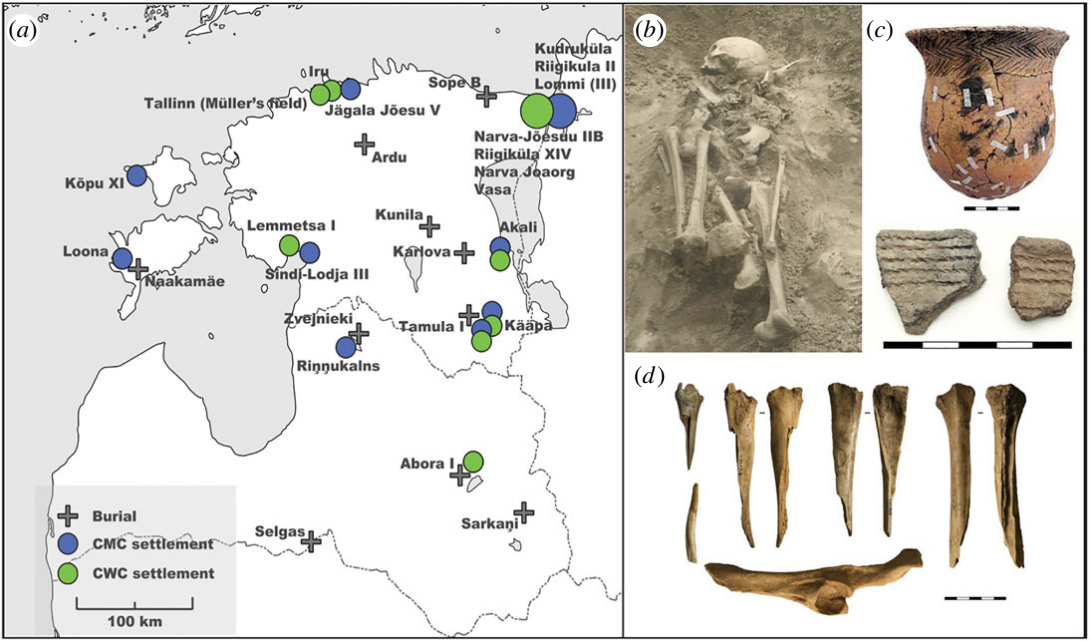
(*a*) Map of the analysed sites in this study, and (*b*) an example of a CWC burial Sope B I, Estonia. (*c*) Examples of CWC pottery: a beaker from the Narva-Jõesuu IIB burial ground (Estonia) and two fragments of Estonian-type Corded Ware pots from the Lemmetsa I settlement (Estonia) and (*d*) early domesticates' bones found as grave goods from the Sope B I burial. Note that the Abora I settlement also includes other Porous and Lubāna pottery types besides the CWC vessels.

We use the more general term ‘farming’, rather than ‘agriculture’, referring mostly to animal husbandry, not necessarily including the element of crop cultivation, although in the context of the northeast Baltic early farming seems to mostly rely on stockbreeding. The term ‘domesticates’ indicates the species (both plant and animal) that rely on human input in their growth cycle and survival. The 4th–3rd millennium cal BCE is traditionally classified as Neolithic in the northeast Baltic, whereas the majority of the period is characterized by pottery-using HFG lifeways. By HFG populations, we refer to local forager groups with different regional and/or cultural affiliations: mostly Typical/Late Comb Ware groups (3900–1800 cal BCE, the two ceramic types are often chronologically difficult to separate without direct dating; hence here collated as Comb Ware culture (CMC)), but also the users of regional Porous (3300–1800 cal BCE, a specific pottery group defined in Latvian archaeology from the Lake Lubāns region) and Lubāna (2600–1500 cal BCE) pottery types in Latvia. By CWC (2800–2000 cal BCE), we refer to archaeological complexes which differ from both earlier and contemporaneous ones: individuals interred in burials with a distinctive (mostly) crouched body position and associated grave goods, such as Corded Ware pottery, various stone axes and adzes, flint knives, both domesticated and wild animal bones and tools made from them; but also the users of Corded Ware pottery, which is a clearly distinguishable 3rd millennium cal BCE ceramic type [27] (figure 1).

## 2. Results

### 2.1. Early domesticated animal bone and plant records

Faunal remains from CWC settlements are scarce and highly fragmented, often mixed with preceding and later materials. In Estonia, early domesticated livestock has been mostly identified in CWC burial contexts [12], where they are found either as unprocessed animal remains or worked bones (i.e. tools or their preceding blanks). In Latvia, besides at least one chisel made from an undated sheep/goat bone from Zvejnieki CWC burial no. 137 [28,29], the earliest domesticates have been suggested based on their find context from the 4th and 3rd millennia cal BCE settlements of Zvidze, Kreiči, Sārnate, Abora I, Lagaža and Ein¸i [10,11,30]. However, since all are multi-period sites without direct AMS radiocarbon dates, this evidence remains inconclusive.

**Figure 2.**
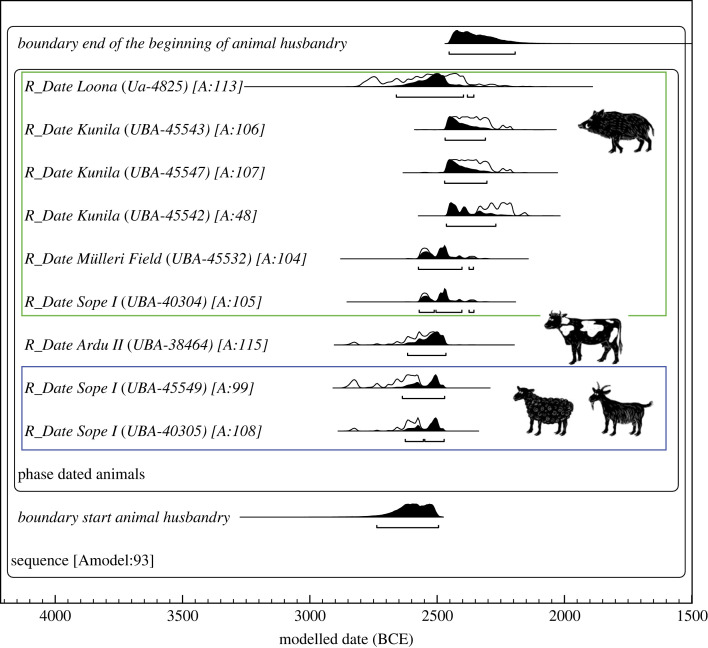
The AMS dates of early domesticates from the northeast Baltic. Calibrated with OxCal 4.4.4. using IntCal20 atmospheric curve [31], all the dates are rounded by 10. For data, table 1.

A range of new radiocarbon AMS dates from a selection of domesticated animal bones from several CWC contexts shows that the introduction of early domesticates in the northeast Baltic took place between 2730 and 2490 cal BCE (modelled starting time, 95.4%; figure 2 and table 1; see also electronic supplementary material, table S1). Although the aurochs (*Bos primigenius*; the ancestor of the domestic cattle) were commonly found across Europe [33], their population in Estonia receded by the 3rd millennium cal BCE, and there are no traces of local domestication of the aurochs or interbreeding with imported domestic cattle [34,35]. Hence the dated specimen must be the domesticated congener. The two sheep/goat samples from the Sope B I burial were subjected to zooarchaeology by mass spectrometry (ZooMS) analysis [36] to clarify the species, but the results were unsuccessful (electronic supplementary material, table S2). These two samples are from different skeletal elements, and their dates represent a single event statistically (2840–2490 cal BCE; *χ*^2^-test d.f. = 1; *T* = 0.113; *T*’(5%) = 3.841, with an overall agreement index (*A*_comb_) 130%), hence they could belong to the same individual. As the taxonomic differentiation between the domestic pig (*Sus domesticus*) and wild boar (*Sus scrofa*) is impeded by bone morphology [12,37], all the suids dating around 2880–2350 cal BCE are cautiously identified as *Sus* sp., although the possibility of domesticated suids has been suggested for the Loona settlement specimen [34].

**Table 1.** An overview of the AMS radiocarbon dates of the earliest domestic livestock in Estonia. All finds, besides Loona, are associated with CWC contexts. Calibrated with OxCal 4.4.4 using IntCal20 atmospheric curve [31], all the dates are rounded by 10.

species	arch. site	site type (burial)	sample ID	skeletal element (U, unworked; P, pretreated tool blank)	^14^C lab no.	^14^C dating BP	cal BCE (95.4%)	reference for ^14^C date
*Ovis aries*/*Capra hircus*	Sope	burial (B I)	AI 2607	Os coxae (U)	UBA-40305	4057 ± 27	2840–2490	this study
*Ovis aries*/*Capra hircus*	Sope	burial (B I)	AI 2607: 2	Radius (P)	UBA-45549	4073 ± 36	2840–2490	this study
*Bos taurus*	Ardu	burial (II)	AI 3499: 66	Os coxae (U)	UBA-38464	4034 ± 41	2850–2460	this study
*Sus* sp.	Sope	burial(B I)	AI 2607: 3	Tibia (P)	UBA-40304	3964 ± 28	2580–2340	this study
*Sus* sp.	Tallinn Müller's Field (Pärnu Rd. 37, 41)	settlement	AI 8244	Scapula (U)	UBA-45532	3970 ± 35	2580–2340	this study
*Sus* sp.	Kunila^a^	burial	AI 3989/AZ-3	Ilium (U)	UBA-45542	3830 ± 29	2460–2140	this study
*Sus* sp.	Kunila^a^	burial	AI 3898/AZ-6	Ilium + ischium (U)	UBA-45547	3889 ± 36	2470–2200	this study
*Sus* sp.	Kunila^a^	burial	AI 3989/AZ-4	Ischium (U)	UBA-45543	3883 ± 32	2470–2210	this study
*Sus* sp.^b^	Loona	settlement	AI 4129	Unknown	Ua-4825	4050 ± 80	2880–2350	Lõugas *et al*. 1996 [32]

^a^Exact burial affiliation unknown.

^b^Specimen absent from the collections.

In terms of domesticated plants, cereal-type grains have not been identified from any CWC context in the northeast Baltic. Single grains of barley (*Hordeum vulgare*) and possibly einkorn (*Triticum monococcum*) have been reported from the Kreiči settlement in Latvia [38]. However, the site was excavated in the 1950s, its contexts are partially mixed with finds from the 5th to 2nd millennium cal BCE, perhaps even later, while the grains are no longer available for direct AMS dating [39]. A barley grain and an impression of another cereal grain have previously been documented in relation to a CWC sherd from the Iru settlement [11,40], but the sherd and the grain are unfortunately lost, making any further analysis impossible. A potential cereal grain imprint, most likely *Hordeum vulgare*, was noted in a Lubāna type sherd from the Abora I settlement during sampling for organic residue analysis reported in this article, but the foodcrust has not been directly dated.

The scarcity of macrobotanical remains has instigated the extensive use of pollen data for reconstructing early cereal cultivation in the northeast Baltic [10,11,41]. Cereal-type pollen grains identified as unspecified cereal (*Cerealia*-type), barley (*Hordeum*-type), wheat (*Triticum*-type) and oat (*Avena*-type) have been previously noted from a few sites from the 7th millennium cal BCE (electronic supplementary material, table S3). Yet, the overall number of samples (and sites) reporting finds of cereal-type pollen remains low up until the beginning of the 4th millennium BCE. An abrupt increase in both sample and site frequency and overall number of cereal-type pollen grains is visible in both Estonia and Latvia from *ca* 3500 cal BCE onwards, whereas in the following millennium the pollen evidence stabilizes or even shows some regression [42]. Gradual increase of open landscapes together with slightly growing evidence of cereal-type pollen finds has been recorded from the beginning of the 2nd millennium cal BCE onwards [41,43].

However, several methodological peculiarities like the uncertainties of dating, pollen identification and estimating pollen source area [39,44,45] must be considered. Most importantly, pollen taxonomy is not directly compatible with conventional botanical taxonomic precision, and one pollen type can include pollen from plants belonging to different species, families or sometimes even genera [46]. Most cereals commonly grown in Europe produce pollen identifiable to the level of pollen type—hence the *Hordeum*-type, *Triticum*-type, *Avena*-type and *Cerealia*-type identifications—and all these types include some species of wild grasses [42,47,48]. Among such wild grass species are several members of the grass family (e.g. *Elymus arenarius*, *Glyceria fluitans*, *Bromus inervis*) common in the northeast Baltic, which produce pollen grains with similar size and/or surface structure to those of the cereals. Hence, arguing for early crop cultivation based solely on pollen findings remains uncertain, and we currently lack supportive evidence from other proxies to substantiate that domesticated plants were part of the main subsistence strategy in the 3rd millennium cal BCE.

### 2.2. Stable isotopes from human bone collagen

The collagen stable isotope analysis (SIA; raw data reported in electronic supplementary material, table S5) contains all the individuals directly dated to the 3rd millennium cal BCE from Estonia (*n* = 10) and Latvia (*n* = 17). Besides these, the Abora I site includes directly dated individuals (*n* = 13), 20 specimens found as loose human bones (*n* = 11) and intact burials (*n* = 9) all found from the settlement layer dated to *ca* 3400–1900 cal BCE [49,50]. The dataset also entails an infant and adult female from the Selgas double grave, and burial 307 from Zvejnieki due to their characteristic traits for CWC burials. This results in a total of 49 individuals, with either single or multiple skeletal elements being analysed, and thus represented by 65 samples of both adult and non-adult values. Since the collagen yield together with carbon and nitrogen percentage inclusion was inconsistently reported in previous studies, we use the C : N ratio falling between 3.1 and 3.5 in this study (electronic supplementary material, table S5) as a quality criterion for collagen preservation [51,52].

**Figure 3.**
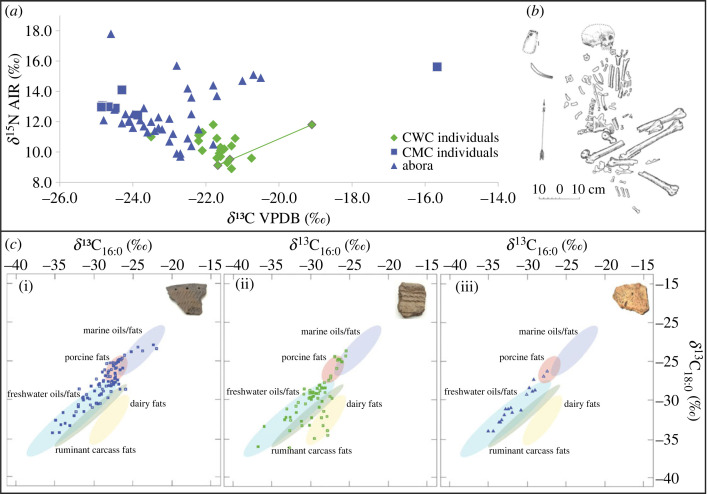
Summary of biomolecular dietary results from the 3rd millennium cal BCE northeast Baltic. (*a*) plot of human bone collagen stable isotope data, (*b*) with an example of a typical CWC burial, Ardu I. Note that the Ardu I individual is displayed with connected data points showing intra-individual dietary change (the higher *δ*^13^C and *δ*^15^N values are from adulthood). Data derive from this study and previous publications [12,18,32,50,53–59]. (*c*) *δ*^13^C values of mid-chain-length fatty acids (C_16:0_ and C_18:0_) extracted from Estonian and Latvian (pre-)3rd millennium cal BCE CMC (i), CWC (ii) and other 3rd millennium cal BCE regional wares from Abora I (iii) with full aquatic biomarkers indicated by filled symbols. The reference ranges presented in C (i-iii), calculated at 68% confidence, were derived from data obtained from the tissues of modern animals, mostly from the Baltic region [60–62].

The human bone collagen SIA values display considerable heterogeneity among the 3rd millennium cal BCE populations (figure 3*a*), with values ranging from −24.9‰ to −15.7‰ for *δ*^13^C, and +8.9‰ to +17.8‰ for *δ*^15^N. The statistical comparison of CMC, CWC and Abora I SIA values shows significant differences (Mann–Whitney pairwise test all *p*-values < 0.05), but due to the small number of CMC samples this must be taken with caution, whereas the difference holds for the Abora I and CWC individuals. The CWC burials have a narrower range of values (*δ*^13^C range −23.5‰ to −19.1‰, *δ*^15^N + 8.9‰ to +11.8‰; see electronic supplementary material, table S4), which is indicative of protein intake from terrestrial sources. The consumption of terrestrial foods is indirectly evidenced by the overlapping paired AMS dates of terrestrial animals and humans from the Ardu II and Sope B I burials (electronic supplementary material, figure S1), showing that humans do not have apparent ages caused by a reservoir offset. Multiple samples are available for three CWC individuals—Ardu I, Kunila I and Sope B I—which permits us to track the dietary protein intake changes in an individual's lifetime. For Sope B I and Kunila I, the dietary change is negligible (less than 1‰), but Ardu I displays elevated *δ*^13^C and *δ*^15^N values in adulthood (based on comparing SIA results from molars and the rib; figure 3*a*).

In contrast to the CWC individuals, there is considerable intra-site variability in the SIA values among the 33 individuals from Abora I. Here the *δ*^13^C values range from −24.8‰ to −20.5‰, and *δ*^15^N from +9.7‰ to +17.8‰, whereas the remarkably high *δ*^15^N value (+17.8‰) from burial 8 l belongs to a non-adult aged 1 ± 2 months [50] and is indicative of weaning. There is no significant chronological or burial practice (crouched versus supine position versus loose human bones) based difference in the Abora I cohort (Mann–Whitney pairwise test *p*-values ranging from 0.14 to 0.98). From the CMC group, the Tamula individuals had higher *δ*^15^N values and lower *δ*^13^C values indicating a reliance on freshwater resources, whereas the Tamula XXII individual demonstrated low lifetime intra-individual dietary changes. The HFG female from Naakamäe (Saaremaa island) with a maximum date range between 2910 and 2490 cal BCE (reservoir effect (RE) corrected, 95.4% probability [32,53]), stands out for her high *δ*^13^C and *δ*^15^N values, being the only individual in our dataset with a clear focus on marine foodstuffs.

### 2.3. Pottery lipids

A total of 178 samples from CMC and CWC pottery from 18 sites in Estonia and Latvia were subject to organic residue analysis (ORA). Of these, 111 samples are from CMC and 67 from CWC vessels. This dataset is further supplemented by 15 Porous and six Lubāna-type ceramic samples from Latvia, resulting in a total of 199 samples, of which 113 are reported here for the first time. The biomarker and isotope results of ORA from all samples are reported in electronic supplementary material, table S6, whereas the results from previous publications [25,63,64] were included in the dataset only if both biomarker and compound specific isotope data was available.

To trace early farming through ORA, *δ*^13^C measurements from the two major fatty acids, C_16:0_ and C_18:0_, are highly informative for distinguishing freshwater and marine organisms, porcine and ruminant fats, and most importantly dairy products [65–67]. As domesticated and wild ruminants are isotopically inseparable, the detection of dairy fats remains the major indicator of exploiting domesticated ruminant species and their products.

A Mann–Whitney *U*-test for the isotopic measurements of the *δ*^13^C_16:0_ and *δ*^13^C_18:0_ fatty acids in the CMC (*n* = 93) and CWC (*n* = 64) samples shows differences between the two groups (*p*-value < 0.029 for both), with the average values for *δ*^13^C_16:0_ and *δ*^13^C_18:0_ ranging between −28.6 ± 2.5‰ and −28.0 ± 2.6‰ for the CMC, and −29.4 ± 2.3‰ and −29.8 ± 3.0‰ for the CWC samples. The *δ*^13^C results (figure 3*c*) from CWC, show the clear inclusion of dairy products in seven (potentially eight) CWC vessels, being the earliest evidence of dairy fats in the northeast Baltic. This number should be considered as a minimum, as mixing dairy with other products might result in isotopic values outside the dairy ellipses, also into the ruminant region [68]. Notably, all the dairy samples are from the north and northeast part of Estonia, with none from the inland sites sampled. There is one sample from Abora I plotting between the dairy and ruminant ellipses, but it also includes the full suite of aquatic biomarkers, which might hint at mixing dairy products with other substances with more depleted *δ*^13^C_16:0_ values.

Despite the first detection of dairy fats in this region, aquatic biomarkers from either marine or freshwater organisms indicate that different aquatic products were processed in both CMC (40 samples out of 93) and CWC vessels (22 samples out of 64) as well as in other regional wares (15 samples out of 18; figure 3*c*). These specific biomarkers include *ω*-(*ο*-alkylphenyl)alkanoic acids with carbon atoms ranging from C_16_ to C_20/22_, formed during the heating of long-chain polyunsaturated fatty acids of aquatic organisms, together with one or more of the isoprenoid fatty acids (phytanic, pristanic and 4,8,12-trimethyltridecanoic (TMTD)) [69,70], with further support by the ratios of 3S,7R,11R,15-phytanic acid (SRR) and 3R,7R,11R,15-phytanic acid (RRR) diastereomers [71]. Hence we have clear evidence for initial dairy production/storage, but also for the consumption of aquatic substances, suids and ruminants throughout the 3rd millennium cal BCE.

## 3. Discussion

Comparison of different dietary proxies—zooarchaeological, archaeobotanical, SIA and ORA—provides a more cohesive picture of the arrival, nature and spread of early farming in the northeast Baltic. In contrast to the northwest Baltic [72], the scarce macrobotanical and pollen data show an absence of considerable crop cultivation in the northeast Baltic during the 3rd millennium cal BCE. Plant consumption here was rather tuned towards wild species [16,73], although tentative experimenting with crop cultivation cannot be entirely excluded [74]. The oldest directly dated cereal grains in the northeast Baltic are from the 2nd millennium cal BCE [39,72,75,76], while crop cultivation at that time is also supported by continuous *Cerealia* pollen records [77,78] and the establishment of the first field systems [21].

Early farming in the northeast Baltic was based on animal husbandry, pioneered by CWC communities who introduced domesticates during the first half of the 3rd millennium cal BCE, and is comparable to the dates for early domesticates from the southeast Baltic [79]. In Finland, the earliest cattle find is from the 2nd millennium cal BCE [80], although a goat hair found from the Perttulanmäki CWC burial might also support an earlier arrival time of domesticates [81]. However, domesticated animals start to dominate the faunal record in the northeast Baltic only around 1000 cal BCE [12,30,34,82].

The introduction of domesticates is unlikely to have transformed the economies of everyone living in the northeast Baltic during the 3rd millennium cal BCE. The individuals afforded CWC burial form an isotopically narrow and homogeneous group (figure 3*a*) most probably relying on terrestrial resources, similar to CWC individuals from Lithuania and Central Europe [2,83]. By contrast, the other contemporaneous human remains display very diverse foodways (figure 3*a*) from strongly aquatic diets, mixed aquatic/terrestrial diets and more terrestrial orientated diets.

At the finer scale of culinary practices, pottery ORA shows the combination of domesticated, wild terrestrial and aquatic resources among the CWC populations (figure 3*c*). Dairy was identified in seven CWC samples (*ca* 10% of analysed CWC samples) from four coastal/estuarine sites, whereas in at least two of those the consumption of other (wild) resources, including aquatic, is evident. Hence CWC populations in the northeast Baltic were relying on a mixed economy with parallels from Lithuanian and Finnish 3rd millennium BCE contexts [6,24,26,84]. Similar tendencies have been also highlighted in the development of early farming in the northwest Baltic [85–87] and Central Europe [88].

The variations in 3rd millennium cal BCE subsistence systems outlined in this study might have different analytical, regional and socio-cultural explanations. The pottery ORA reflects the dietary practices of wider communities, while SIA shows the dietary history of single individuals (CWC burials) preserved to this day. We can hypothesize that the discrepancy between the CWC vessels' ORA (mixed economy) and CWC burial SIA results (mostly terrestrial diet) might point to different dietary availability and/or preferences among different CWC groups.

The individuals from the traditional CWC burials display a rather homogeneous range of SIA values, the vast majority of domesticated animal bones have been found in CWC burial contexts, while dairy products are detected in CWC vessels from a limited number of coastal/estuarine sites. Therefore, given that the pottery ORA and human bone collagen SIA data are contemporaneous, one possible explanation is that in the introduction phase, domesticated species and their secondary products might have been more accessible to certain subgroups of CWC people, who were afforded a specific burial rite. Alternatively, we might be witness to analytical constraints, as it must be acknowledged that bulk SIA cannot trace the limited consumption of aquatic foods [89]. The inclusion of smaller proportions of aquatic products into the diets of the individuals in CWC burials could be refined with compound specific SIA of amino acids from bone collagen in the future [90]. Finally, the discrepancy between the pottery ORA and human SIA values might also derive from specific culinary practices, i.e. aquatic products being preferably processed in ceramic vessels, whereas a wider range of other foodstuffs were both procured and consumed without the use of ceramic containers.

Nevertheless, CWC pottery ORA shows that the abundant wild resources of northern latitudes offered optimal conditions for practising a mixed economy during the Mid-Holocene. Indeed, the exploitation of wild resources was common among CWC populations from the very beginning [91], with several CWC burials in the northeast Baltic including wild animal bones as well [12,79,92]. The Ardu I CWC individual whose burial customs and childhood diet follow the characteristic CWC traditions, serves as an intriguing example of dietary conversion from fully terrestrial to the mixed economy and a shift towards consuming aquatic, possibly marine resources, in his adulthood (figure 3*a*). The CWC early farmers were adaptive to different subsistence strategies, which might have facilitated their movement into northerly latitudes, whereas the natural conditions in the northeast Baltic probably provided favourable conditions for larger inclusion of forager subsistence elements into their livelihood.

There was no transition from HFG to farming in the 3rd millennium cal BCE northeast Baltic. The introduction of domesticates had little if any impact on local HFG groups, who continued to rely on wild economies and show no signs of early farming through ORA of pottery or SIA of human bone (figure 3). This proves the coexistence of parallel worlds, where farming and/or mixed economy-based CWC groups resided together with the foragers for nearly a full millennium. Such a socio-economic divide is perhaps also reflected by the ancient genomic data: the Tamula (south Estonia) and Naakamäe 3rd millennium cal BCE individuals following forager diets are affiliated with HFGs [17], whereas the CWC populations show different genetic ancestry, originating from/related to the steppe region [18,56]. Similar cultural but also genetic divergence between foragers and early farmers has been established in other parts of Europe across the so-called Neolithic transition [93–96]. However, the proportions of CWC and local CMC populations among the 3rd millennium cal BCE in the northeast Baltic need further investigations to determine the extent of population turnover prior to *ca* 2000 cal BCE [19].

The northeast Baltic 3rd millennium cal BCE data provides important insights for acknowledging the complexity for HFG–farmer transitions. There was no single or linear pathway in the change of subsistence systems, and we are very far from the hierarchical and ‘all-or-nothing’ conceptualization when it comes to early farming practices [97–99]. In fact, the whole concept of ‘transition’ is erroneous in the northeast Baltic context, as local HFGs remained true to their forager lifeways, which existed in parallel with (segregated) farming pioneering, yet mixed economy CWC populations. There is probably no total replacement of foraging by farming as the major source of food production in northern latitudes (beyond the 56th parallel north), and future research should rather address the economic dialogues between the wild and domesticated, as well as tackle the prolonged adaptation and experimenting processes with several success–failure episodes over the following millennia.

## 4. Methods

### 4.1. Organic residue analysis: sample preparation

Majority of samples were prepared and analysed at the University of Tartu Archemy laboratory and University of York BioArCh facility. Food crust adhered to pottery surfaces were removed using clean scalpels. Ceramic powder samples were drilled using clean drill bits, discarding the upper surface powder to avoid contamination. Lipids were extracted and derivatized (methylated) from *ca* 1 g of ceramic powder and 20 mg of foodcrusts using acid-catalysed methylation following previously reported procedure [61]. The samples were analysed with gas chromatography–flame ionization detector (GC-FID) for lipid quantification, gas chromatography–mass spectrometry (GC-MS) for general biomolecular characterization, and GC-MS in selected ion monitoring (SIM) mode targeting aquatic biomarkers and GC–combustion–isotope ratio mass spectrometry (GC-C-IRMS) to determine the *δ*^13^C values of C_16:0_ and C_18:0_ fatty acids.

A subset of samples (sample nos. 186–199 reported in electronic supplementary material, table S6) was analysed at the University of Bristol, School of Chemistry using the previously reported simultaneous extraction and methylation protocol [100], followed by the analysis with high temperature (HT) GC-FID, GC-MS and GC-C-IRMS.

### 4.2. Gas chromatography–flame ionization detector analysis

GC-FID analysis was conducted at the York BioArCh facility (University of York) using Agilent 7890 B Series GC and DB1-HT polyimide-coated fused silica column (15 m × 320 µm × 0.1 µm; J&W Scientific, Folsom, CA, USA). Injected sample size was 1 µl. The splitless injector was used at 300°C with helium carrier gas. The temperature was set at 100°C for 2 min, with the gradient of 20°C min^−1^ up to 325°C maintained for 3 min. Data were acquired using ChemStation software.

In Bristol, the analyses were undertaken using an Agilent Technologies 7890A GC system and 100% dimethyl polysiloxane-fused silica column (15 m × 320 µm × 0.1 µm; Restek, Rxi-HT). Injected sample size was 1 µl. The on-column injector was used with helium carrier gas. The temperature programme was set at 50°C for 2 min, followed by a gradient of 10°C min^−1^ up to 350°C, followed by a 10 min isothermal hold. Data were acquired using HP ChemStation software (Rev. B.03.02 [341] Agilent Technologies).

### 4.3. Gas chromatography–mass spectrometry analysis

GC-MS was conducted at the Institute of Chemistry (University of Tartu) with Agilent 7890A Series gas chromatograph and Agilent 5975C Inert XL mass-selective detector with a DB5-MS (5%-phenyl)-methylpolysiloxane column (30 m × 0.25 mm × 0.25 µm). Injected sample size was 1 µl. The splitless injector and interface were maintained at 300°C and 280°C, respectively, helium was used as the carrier gas at a constant flow. The GC column was inserted directly into the ion source of the mass spectrometer. The temperature programme was set as follows: 50°C for 2 min, thereafter a gradient of 10°C min^−1^ up to 325°C and kept there for 14.5 min. Compounds were identified with Agilent Chemstation software using also NIST mass spectral library.

Additional dedicated GC-SIM method was used for detecting aquatic biomarkers as reported in previous studies [61,101]. For this GC-MS equipped with a DB-23 (50%-cyanopropyl)-methylpolysiloxane column (60 m × 0.250 mm × 0.25 µm; J&W Scientific, Folsom, CA, USA) was used. Injected sample size was 1 µl. The oven temperature was set at 50°C for 2 min with an increase of the temperature up to 100°C (10°C min^−1^). Thereafter, the temperature was raised by 4°C min^−1^ to 140°C, then by 0.5°C min^−1^ to 160°C and, finally, by 20°C min^−1^ to 250°C where it was maintained for 10 min. The carrier gas used was helium with a flow rate of 2.4 ml min^−1^. The SIM mode was set to target characteristic aquatic biomarkers' ions groups: *m/z* 74, 87, 213, 270 for 4,8,12-trimethyltridecanoic acid (TMTD), *m/z* 74, 88, 101, 312 for pristanic acid, *m/z* 74, 101, 171, 326 for phytanic acid and *m/z* 74, 105, 262, 290, 318, 346 for the detection of *ω*-(*o*-alkylphenyl) alkanoic acids of carbon lengths C_1__6_–C_22_ (APAA_16–22_). Additionally, two phytanic acid diastereomers (3S,7R,11R,15-phytanic acid or SRR and 3R,7R,11R,15-phytanic acid or RRR) were separated for the calculation of the percentage of SRR in total phytanic acid (SRR%) by integrating the *m/z* 101 ion [71].

GC-MS analysis in Bristol was performed using a Thermo-Finnigan Trace MS and introduced via a PTV injector set to splitless mode onto an HP-1 fused silica capillary column (100% dimethylpolysiloxane, 50 m × 0.32 mm × 0.1 µm; Agilent). Injected sample size was 1 µl, and helium was used as a carrier gas at a constant flow. The temperature programme was set as follows: 50°C for 2 min, thereafter gradient of 10°C min^−1^ up to 300°C where it was held for 10 min. The MS was operated in electron ionization (EI) mode at 70 eV, and acquired between *m/z* 50 and 650 at 1.7 scans s^−1^ in full scan mode. Data were acquired and analysed using XCalibur (v. 1.2).

### 4.4. Gas chromatography–combustion–isotope ratio mass spectrometry analysis

GC-C-IRMS analysis was conducted on acid-extracted (methylated) samples to determine the ^13^C/^12^C ratio in two most abundant fatty acids (C_16:0_ and C_18:0_). At the York BioArCh facility, the samples were analysed with two instruments: Delta V Advantage isotope ratio mass spectrometer (Thermo Fisher, Bremen, Germany) linked to a Trace Ultra 1310 gas chromatograph (Thermo Fisher) with a GC Isolink II interface (CuO combustion reactor held at 850°C); and with Isoprime 100 (Isoprime, Cheadle, UK) linked with Agilent 7890B Series Gas Chromatograph (Agilent Technologies, Cheadle, Cheshire, UK) with Isoprime GC5 interface (Isoprime Cheadle, UK, with CuO combustion reactor held at 850°C). Parallel acquisition of the molecular data was achieved by deriving a small part of the flow to an ISQ mass spectrometer (Thermo Fisher) and Agilent 5975C inert XL mass-selective detector (MSD) equipped with a quadrupole mass analyser (Agilent technologies, Cheadle, Cheshire, UK), respectively.

All fatty acid methyl esters (FAMEs) samples were diluted with *n-*hexane, and subsequently 1 µl of each sample was injected into a DB-5MS UI fused-silica column (PN 122-5562UI; 60 m × 250 µm × 0.25 µm; J&W Scientific technologies, Folsom, CA, USA). The split/splitless injector was operated in splitless mode. The temperature was set at 50°C for 0.5 min and raised by 25°C min^−1^ to 175°C, then raised by 8°C min^−1^ to 325°C where it was held for 20 min. Ultra-high-purity-grade helium was used as the carrier gas at constant flow (2.0 ml min^−1^). All samples were measured in duplicates.

Eluted products were combusted to CO_2_ and ionized in the mass spectrometer by electron impact. Ion intensities of *m/z* 44, 45 and 46 were monitored in order to automatically compute the ^13^C/^12^C ratio of each peak in the extracts. Computations were made with Isodat (v. 3.0; Thermo Fisher) and IonOS/LyticOS software (Isoprime, Cheadle, UK) and were based on comparisons with a repeatedly measured standard reference gas (CO_2_). The results from the analysis are reported in parts per thousand (‰) relative to an international standard (V-PDB) and corrected for the carbon atom added during methylation using a mass balance equation [102].

At Bristol the samples were analysed with Isoprime 100 (Isoprime, Cheadle, UK) linked with Agilent 7890A Series GC (Agilent Technologies, Cheadle, Cheshire, UK) via an Isoprime GC5 interface (Isoprime Cheadle, UK; with furnace temperature of 850°C). The FAMEs were diluted with *n-*hexane and injected (1 µl) into an HP-1 fused silica column with a dimethyl polysiloxane stationary phase (50 m × 0.32 mm × 0.17 µm; Agilent). The temperature programme was set at 40°C for 2 min and raised 10°C min^−1^ to 300°C where it was held for 10 min. Helium was used as a carrier gas at constant flow (2.0 ml min^−1^). All analyses were conducted in duplicate. Following conversion to CO_2_ over copper oxide pellets, ion intensities of *m/z* 44, 45 and 46 were monitored to determine ^12^C/^13^C, relative to the repeatedly measured standard reference CO_2_ gas. Instrument performance was determined using an in-house FAME mixture (C_11:0_, C_13:0_, C_16:0_, C_21:0_, C_23:0_) with known *δ*^13^C values, with an instrument precision of ±0.3‰, determined via repeated analysis between samples. Data processing was carried out using IonVantage (v. 1.5.6.0, Isoprime). *δ*^13^C values are reported in parts per thousand (‰) relative to the international standard (V-PDB), and were corrected for the methyl group added during derivatization via a mass balance equation [102].

### 4.5. Zooarchaeology by mass spectrometry analysis

ZooMS analysis was carried out following previously published protocols [103,104]. Bone chips were demineralized in 0.5 M HCl and rinsed in 50 mM ammonium bicarbonate. The demineralized bone chip was then gelatinized at 65°C for 1 h and the resulting supernatant was treated with 0.4 µg trypsin (Thermo Scientific Pierce Trypsin Protease). Enzymatic digestion took place at 37°C for 18 h. The incubated samples were concentrated and desalted using C18 ZipTips (Thermo Scientific Pierce C18 Tips) and eluted in a final solution of 50 µl of 50% acetonitrile and 0.1% trifluoroacetic acid (TFA). Then 0.5 µl of the resulting solution was mixed with 0.5 µl of *α* cyano-4-hydroxycinnamic acid solution (10 mg ml^−1^ in 50% acetonitrile and 0.1% trifluoroacetic acid) and allowed to crystallize.

Samples were analysed at the Max Planck Institute for the Science of Human History using a Bruker AutoFlex LRF Speed. The resulting spectra were peak picked with a signal to noise ratio of 3.5 after baseline correction, smoothing and deisotoping with the default parameters and analysed with flexAnalysis 3.4 (Bruker Daltonics) and mMass software [105]. The spectra were compared against a reference library of known peptide markers [36,103,106,107]. The resulting spectra are provided (see electronic supplementary material, Data ZooMS files) and peptide markers are reported following standard nomenclature [108] (see electronic supplementary material, table S2).

### 4.6. Collagen stable isotope analysis

The SIA results presented in this paper derive mostly from published materials and thus are conducted at different laboratories with different pretreatment protocols for collagen extraction. The extraction methods and hardware used for analysis are described in the papers referred to in electronic supplementary material, table S5.

The samples analysed in this study (burials from Sope, Ardu, Kunila) were analysed at the Archemy Lab, University of Tartu (UT). Collagen was extracted using the modified Longin method following the previously reported procedure [109]. Bone/tooth dentine samples were demineralized in 0.25 M hydrochloric acid (HCl) at room temperature for 48 h. The demineralized samples were rinsed with deionized water, which was replaced with 0.01 M HCl solution, to be placed in the oven (58°C) for 16 h for gelatinization. The resulting solution was filtered with Whatman nitrocellulose membrane filters with pore size 5 µm to remove any insoluble residue and freeze-dried. Stable carbon (*δ*^13^C) and nitrogen (*δ*^15^N) isotopic values were measured with an automated carbon and nitrogen elemental analyser isotope ratio mass spectrometer (EA-IRMS) in the Department of Geology, UT. Samples were weighed as duplicates into tin capsules (approx. 1.0–1.2 mg) and combusted in a Thermo Flash HT EA with introduction of separated N_2_ and CO_2_ to a Delta V plus via a ConFlo IV interface. The data were calibrated against international standards from IAEA (for nitrogen IAEA N-1, *δ*^15^N_AIR_ = + 0.4‰, IAEA N-2, *δ*^15^N_AIR_ = + 20.3‰, USGS25, *δ*^15^N_AIR_ = −30.4‰, and for carbon IAEA CH3, *δ*^13^C_VPDB_ = −24.72‰, and IAEA CH6, *δ*^13^C_VPDB_ = −10.449‰). The results are expressed using the delta notation in per thousand (‰) [110] relative to the international marine limestone VPDB standard for carbon and AIR for nitrogen. The long-term stability error for the isotope ratio measurements determined from repeated measurements of international and laboratory standards was better than ±0.2‰ (1 s.d.) for nitrogen, and ±0.1‰ (1 s.d.) for carbon.

### 4.7. Accelerator mass spectrometry dating

Approximately 1 g of sample was removed from the animal bones and submitted to AMS radiocarbon dating at the CHRONO Centre in Queen's University Belfast. The obtained AMS dates were calibrated with the OxCal 4.4.4., using the IntCal20 atmospheric calibration curve [31] and rounded by 10.

## Data Availability

The data are provided in electronic supplementary material [111].
